# LILBID-MS: using lasers to shed light on biomolecular architectures

**DOI:** 10.1042/BST20190881

**Published:** 2022-06-13

**Authors:** Nils Hellwig, Janosch Martin, Nina Morgner

**Affiliations:** Institute of Physical and Theoretical Chemistry, Goethe University Frankfurt/Main, Max-von-Laue-Str. 9, 60438 Frankfurt/Main, Germany

**Keywords:** ionization, LILBID-MS, mass spectrometry, membrane mimics, membrane proteins

## Abstract

Structural Biology has moved beyond the aim of simply identifying the components of a cellular subsystem towards analysing the dynamics and interactions of multiple players within a cell. This focal shift comes with additional requirements for the analytical tools used to investigate these systems of increased size and complexity, such as Native Mass Spectrometry, which has always been an important tool for structural biology. Scientific advance and recent developments, such as new ways to mimic a cell membrane for a membrane protein, have caused established methods to struggle to keep up with the increased demands. In this review, we summarize the possibilities, which Laser Induced Liquid Bead Ion Desorption (LILBID) mass spectrometry offers with regard to the challenges of modern structural biology, like increasingly complex sample composition, novel membrane mimics and advanced structural analysis, including next neighbor relations and the dynamics of complex formation.

## Introduction

Proteins and their complexes play an important role in many biological systems. Out of these systems, membrane proteins pose a special challenge due to their high hydrophobicity. The structural analysis of these systems is challenging for many analytical methods including mass spectrometry (MS).

**Figure 1.**
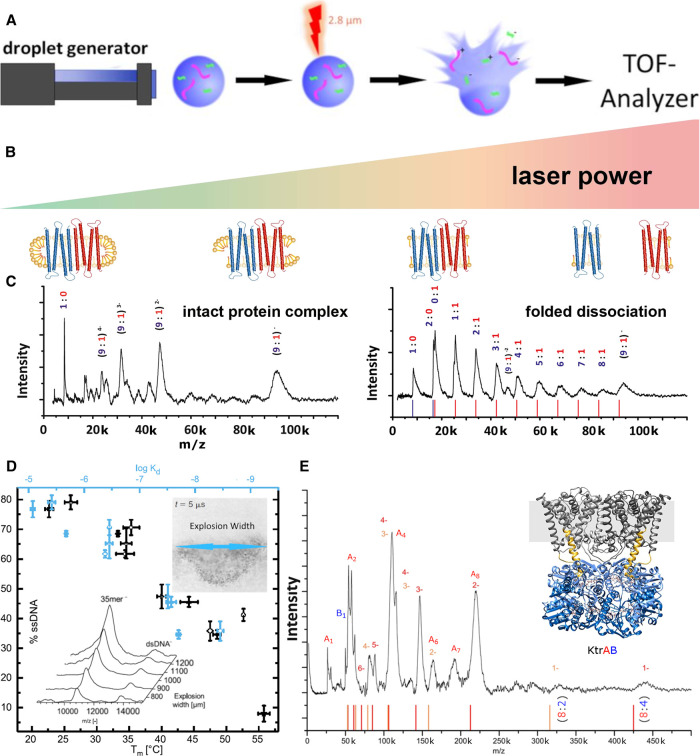
(**A**) Schematic depiction of the LILBID process. A microdroplet of the sample solution is irradiated with a laser pulse leading to the release of the sample ions in their native charge state. (**B**) Influence of the desorption laser power: lower laser power leads to more intact complexes. (**C**) The c-ring of the sodium-driven Na^+^-F_1_F_O_-ATPase of *Acetobacterium woodii* is unusual, as it contains two different c-subunits (c_2/3_ and c_1_). Low laser power releases a charge distribution of intact rings (left), while under high laser power (right), the c-ring is dissociated into a series of fragments, which allowed to determine the stoichiometry to be 9 : 1 [17, 18]. (**D**) Correlating LILBID laser power with the resulting dissociation of DNA double strands allows the prediction of dissociation constants. The inset shows signal intensity of double stranded DNA and the dissociated single strand in dependence of different LILBID laser intensities. The explosive expansion of the sample droplet is visualized and the width of the resulting particle cloud is calculated as a measure for the energy absorbed by the droplet [19]. (**E**) LILBID mass spectra of the K^+^ channel KtrAB allow for the determination of the stoichiometry without hampering effects of even high alkali metal ion concentrations, which would make analysis with nESI challenging [20].

A key step in a successful MS analysis is the transfer of the analyte molecules from the condensed phase to the gas phase. If investigating proteins and their complexes, this transfer will always disrupt the complex from its environment, so the goal in native MS is to facilitate this in a manner as soft as possible. The most commonly used ionization technique for the analysis of membrane protein complexes today is nano-Electrospray Ionization (nESI). A good overview of the applicability of nESI can be found in recent reviews on the subject [1–14] and is beyond the scope of this review. Laser Induced Liquid Bead Ion Desorption (LILBID) MS is another soft ionization technique, which was introduced in 2006 [15] and was among the first MS methods to be applied to membrane proteins [16]. In LILBID, the sample is introduced into the mass spectrometer in small droplets (50 µm diameter) of sample solution and is irradiated by a mid-IR laser with pulse energies varying from 10 to 23 µJ, which is absorbed by the water molecules surrounding the analyte. The laser pulse triggers an explosive expansion of the droplet and the sample ions are released into the gas phase without any additional active charging. This process is depicted in Figure 1A.

LILBID MS is until now not as widely used, due to the lack of commercial availability and its instrumental requirements, albeit some similar approaches are in use [20–25]. However, LILBID has shown its potential, which is especially useful in areas where routine nESI analysis might reach its current boundaries. This includes the special applicability for membrane proteins, which will be the subject of this article. Modern mass spectrometry can nowadays deliver more insights into a system of interest, such as a soluble or membrane protein complex, than just its mass. Areas of high relevance to be investigated are for example the determination of complex stoichiometries and binding events as well as factors influencing these; the structural analysis of complexes e.g. next neighbor relations and pathways in complex formation. A topic which becomes more and more favored for the investigation of membrane proteins not only for MS applications is the employment of sophisticated membrane mimics e.g. nanodiscs and SMALPS, to avoid destructive influences often concomitant with their purification.

## Sample conditions

The key requirement for the successful structural analysis of a protein complex outside of a cell is keeping it in a state as close to native as long as possible. Relevant factors are a buffer system to ensure the physiological pH is preserved and system dependent further additives may be needed to emulate the native environment e.g. NaCl, Mg^2+^-ions, nucleotides or ATP. Membrane proteins additionally require some means of membrane mimic to keep them stable outside of their native cell membrane due to their hydrophobic nature. Certain specific lipids can also be involved in the function of the complex. Each of these factors can have hampering effects on mass spectrometrical analysis of the protein complex, due to challenges such as signal suppression or overlap and peak broadening caused by adducts [26]. Removal of these additives can be essential, especially to answer questions such as the binding of a small molecule or modifications of a protein. Stripping the complexes of interest of such unspecific adducts requires energy, which needs to be transferred into the system without influencing the complexes features of interest. For this reason, volatile buffers like ammonium acetate or carbonate are widely used in native mass spectrometry, but as mentioned above not always sufficient to mimic all relevant features provided by the cell to keep a protein complex in its intact native state [26–31]. LILBID has proven to be able to handle a number of additives in higher amounts compared with other native MS methods, ranging from salts (e.g. NaCl) over buffers (e.g. HEPES) to detergents (e.g. Brij 35) [17].

The key difference between LILBID and nESI regarding the removal of attachments is the mechanism of energy input. In mass spectrometers using nESI ion sources, the most commonly used method is collisionally induced dissociation, where the charged protein complex is exposed to a multitude of collisions with an inert collision gas. In this case, the measure for the energy input is the acceleration voltage used to induce these collisions. LILBID uses an IR laser pulse of a few ns for the release of the sample ions from solution. This energy input happens on a much faster time scale (ns vs µs) than the equivalent energy input via collisions. The pulse energy used here determines the energy input into the protein complex and can be varied from softer to harsher conditions. This causes in a laser energy dependent manner the release of the solvated complex ions, aids the removal of attachments and also allows to dissociate the complex in dependence of interface stabilities to determine the constituting building blocks, next-neighbor relationships and stoichiometries (see Figure 1B and C) [18]. A method that can be used in combination with nESI, which is as well based on a fast transfer of energy and produces similar dissociation patterns is surface induced dissociation (SID) [32–34]. Control over the LILBID laser dependent dissociation process enables the determination of binding affinities, as it was demonstrated using a set of DNA double strands as a model system and analyzing the degree of complex dissociation in relation of transferred laser energy, shown in Figure 1D [19].

The limit of detection and sample consumption are about equal in LILBID and nESI. A directly noticeable difference are the protein charges in the mass spectra. As no active charging occurs during the ionization process in LILBID, proteins generally carry less charges than after ionization with nESI. Both methods perform well for water-soluble protein complexes, which are generally less demanding with regard to requirements for buffer and additives [17].

One reason why a high concentration of additives can hamper nESI-MS, is the ESI inherent droplet shrinking process, which concentrates these additives even more [35]. This effect can be lessened by lowering the initial size of the nESI droplets [36–41], but not overcome completely. In contrast the LILBID laser desorption mechanism sets ions free without prior droplet shrinking, so comparably high starting concentrations of additives can be tolerated for LILBID measurements, albeit they might lead to peak broadening and an increase in observed mass due to attachments. Nevertheless, even mass spectra afflicted with intensive peak broadening due to attachments, can allow for the unambiguous assignment of the LILBID peaks due to their low charge states. An example is shown in Figure 1E; here the potassium channel KtrAB is depicted, a complex consisting of the hydrophobic KtrB and the soluble KtrA subunits. The complex stability required a salt concentration of 40 mM, which could be added without reducing the spectral quality (the buffer contained 25 mM NaCl, 15 mM KCl, 20 mM Tris–HCl pH 8.0 and 1.5 mM Cymal-6). The LILBID spectra revealed the presence of a KtrB_2_A_8_B_2_ and a KtrB_2_A_8_ complex [42]. (see as well Figure 3B for another example of a high salt measurement at 150 mM NaCl).

## Analysis of membrane protein complexes in nanodiscs

While detergents can be a good means to solubilize membrane proteins, they do not mimic all aspects of a membrane to an equal degree. Depending on the system and the question asked, the presence of lipids in a membrane-like arrangement might be a vital prerequisite for meaningful analysis. Nanodiscs are an artificial means of surrounding a membrane protein with a more native-like environment than detergent micelles [43–45]. Nanodiscs consist of a lipid bilayer, which is stabilized by a membrane scaffold protein (MSP), whose length determines the size of the nanodisc. This more rigid environment and the diversity in mass due to the distribution of the number of lipids in one nanodisc can pose a considerable challenge to mass spectrometric analysis. Challenges include the heterogeneity of the lipid discs and the difficulty of stripping the proteins of the surrounding membrane mimic without drowning the signals of interest in lipid cluster signals. This had limited existing nESI studies mostly to the investigation of empty nanodiscs, but with the use of supercharging agents and improving instrumentation, membrane proteins have become accessible using nESI [46–51]. In contrast, LILBID-MS has proven numerous times now to be well suited for the analysis of membrane proteins in nanodiscs without the need for special additives.

Screening experiments of empty nanodics revealed that low desorption laser power leads to broad peaks due to the diversity of lipids inside the nanodiscs. High laser power however produces distinct peaks of MSP monomers and dimers with a small, determinable number of lipids attached. LILBID spectra of protein complexes in nanodiscs can show signals of the complexes, MSP and lipids, as well as combinations. Softer conditions favor transmission of bigger constructs, while harsher conditions reveal all possible dissociation products. These allow to investigate oligomers, as was shown for the first time for protein complexes of known oligomeric states like EmrE (dimer) and KcsA (tetramer) in nanodiscs and applied in several instances since to investigate unknown complexes [52–54]. Assignment difficulties based on too similar sized MSP and proteins can be overcome with the use of isotopic labeling of one or the other [55].

**Figure 2.**
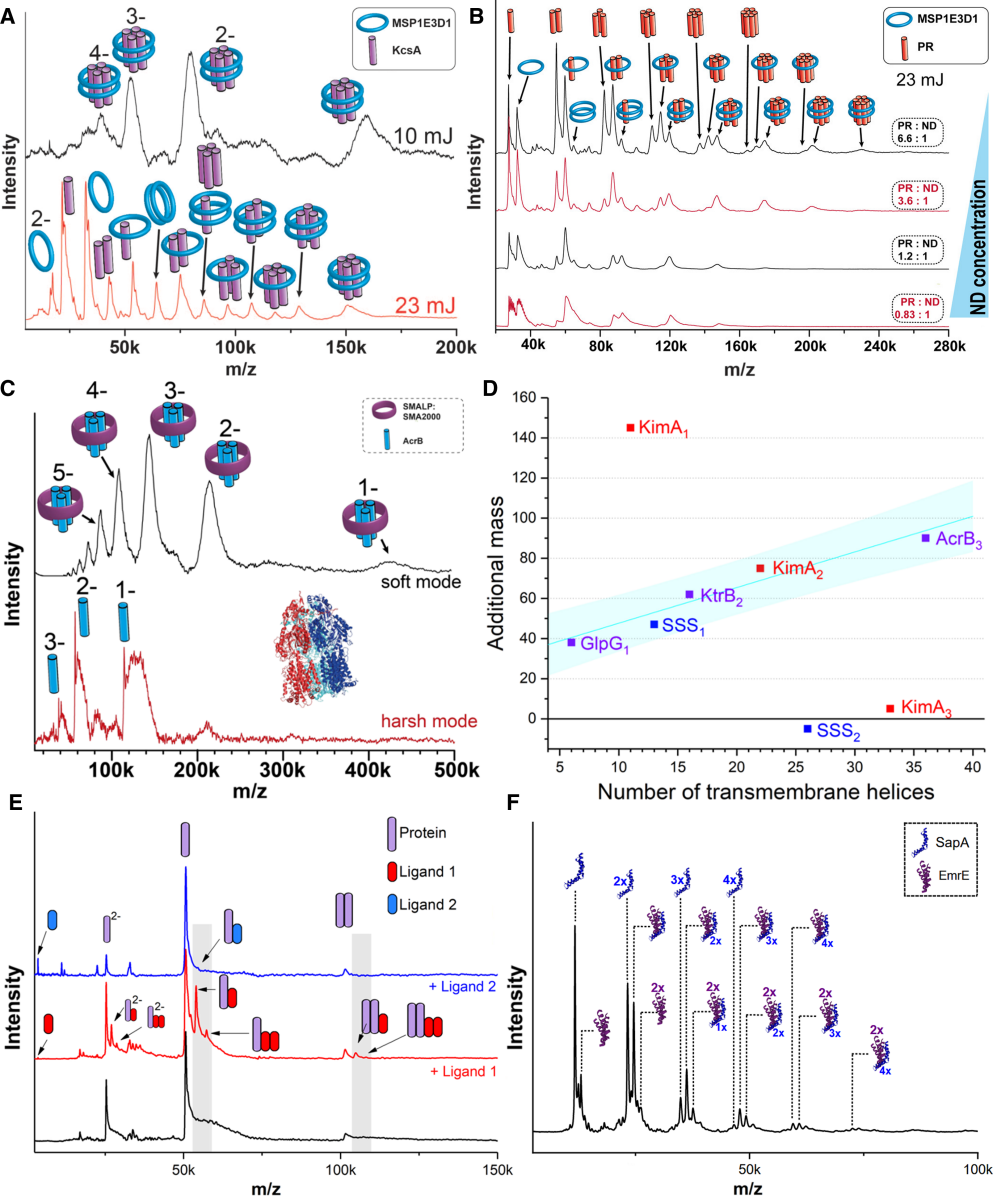
(**A**) The potassium channel KcsA can be detected in its natural tetrameric state after cell-free expression into a nanodisc [56]. Higher laser power leads to the dissociation of the complex, revealing complexes stripped of the MSP, proving that proteins integrated into the nanodisc from stable complexes. This can as well be seen in (**B**) for proteorhodopsin, a large proton pump which is known to form an active hexamer [57]. The protein-to-nanodisc ratio is an important factor in the assembly of the protein complex in the cell-free expression system. The mass spectra show that average protein integration and therewith maximal complex size is reduced at higher nanodisc concentrations [56]. (**C**) Mass spectra of the multidrug efflux pump AcrB measured from SMALPs under low laser power reveal intact trimers plus additional mass stemming from the lipids and polymers surrounding the protein complex. Higher laser power leads to dissociation to monomers, while strongly bound lipids can remain attached [58]. (**D**) The additional mass shift from attached lipids and polymers can be correlated to the number of transmembrane helixes in the complex. This relationship can be used to determine unknown stoichiometries of protein complexes [58]. (**E**) Intact dimers of a model protein can be released from a SMALP while also maintaining specific binding to ligand 1 whereas unspecific control ligand 2 doesn't remain bound (unpublished). (**F**) Saposin-lipid-nanoparticles can also be used for LILBID-MS analysis, in this case the natural dimeric state of multidrug transporter EmrE can be detected in a complex with a varying number of SapA bound to it (unpublished).

The possibility to analyze protein complexes out of nanodiscs allowed as well to understand important factors, which influence successful integration of the proteins into nanodiscs. As this is prerequisite to obtain meaningful results these factors should be generally considered, if working with nanodiscs, such as choosing an MSP of sufficient size for the respective protein complex to avoid restriction of the complex formation process. The choice of lipids and sometimes lipid mixtures forming the bilayer can as well be important to support protein integration into the nanodiscs. Especially for cell-free expression into nanodiscs the ratio of nanodiscs to protein complex is of significance [56]. It allows control over the formed oligomeric state, while at the same time enabling the observation of the complex forming process (See Figure 2A,B).

The controllable environment of a membrane protein in an artificial lipid surrounding offers now the exciting option to investigate structure-function relationships for instance with regard to specific lipids. This allowed for example to understand why the membrane-embedded enzyme MraY, which is involved in the bacterial peptidoglycan biosynthesis, showed differences in the enzymatic activity for the same enzyme from different organisms, *E. coli* and *B. subtilis*. The MS results allowed to correlate the observed differences in activity with lipid dependent oligomerization into the active dimeric state, shedding light of the different structure function relationships of the enzymes from different organisms [55].

This gives a general idea about the employability of artificial nanodiscs (for example in combination with cell-free expression) to determine oligomeric states of active membrane proteins in dependence of functionally relevant factors, such as certain lipids.

## Native MS of membrane proteins in alternative membrane mimics

The above-described nanodiscs allow investigating membrane complexes out of artificial lipid bilayers. A recent, more sophisticated approach to provide a membrane-like environment to membrane complexes is the use of amphiphilic styrene-maleic acid (SMA)-copolymers [59–61]. These can extract membrane proteins directly from their native cell membrane including a ring of annular lipids. These polymer–lipid-combinations - so called SMA lipid particles (SMALPs) or native nanodiscs, allow investigation of the membrane proteins in their native lipid environment, encapsulated directly out of their native cell membrane.

LILBID-MS as well as nESI-MS in combination with a collision cell [62] can be applied to release monomeric proteins of membrane complexes directly from SMALPs, which enables further investigation of these monomers. LILBID additionally allows, as the first, and to our knowledge currently only mass spectrometric method, to determine the oligomeric state of membrane protein complexes from SMALPs either directly (original Data in Figure 2E) or indirectly [58]. The release of the intact protein complex from SMALPs is less straight forward than for nanodiscs, likely due to the strong interactions between the protein and its native lipid environment (see Figure 2C for an example). Nevertheless, a reliable estimation of the mass of the polymer/lipid environment can be obtained in relation to the number of transmembrane helices in the protein complex. This allows the unambiguous determination of a complexes native oligomeric state. (visualized in Figure 2D) The combination of the two methods is also promising for the analysis of interactions between membrane proteins and their native membrane environment, as we observe that proteins dissociated from the SMALPs can retain lipids, from the native cell membrane, which are especially tightly bound. Recent results also prove, that it is not only possible to retain the oligomeric state of a complex during its release from the SMALP, but also to maintain binding of a specific ligand. (Figure 2E) This allows to probe ligand binding by incubating protein complexes in SMALPs with different ligands.

Another new approach at mimicking a membrane are saposin-lipid-nanoparticles, which have similarities to MSP nanodiscs, but employing saposins instead of MSPs to surround the lipids [63, 64]. As the number of saposins forming these discs can vary with the size of the complex, the resulting saposin-lipoprotein nanoparticles are more flexible. LILBID spectra can again be the means to detect protein complexes released from such a nanoparticle, allowing to reveal a complexes stoichiometry. (Figure 2F)

## LILBID for structural analysis

Besides the stoichiometry of homo- and heterogeneous protein complexes, the next-neighbor relationships of the complexes subunits and their binding interfaces are of high interest to understand the structure and function of a complex. The asymmetric dissociation observed in nESI, especially while using CID, based on unfolding of a peripheral subunit, can make it difficult to determine underlying structural information. This effect can be amplified for membrane proteins as usually a higher collisional activation is needed to release the complex from its solubilization agent, be it detergent micelle or one of the more sophisticated mimics [65, 66].

We have found no such effects of detergents for the laser-induced dissociation in LILBID. A direct comparison of both methods was performed for several systems under the same conditions. The most simple system was a homodimeric membrane protein complex consisting almost entirely of transmembrane helices - EmrE solubilized in DDM [17]. Low laser power allows to strip detergents from the complex, releasing the dimer as dominating species for MS analysis. nESI measurements of EmrE in the same buffer conditions allowed under optimal settings the observation of the dimer only as a minor species, demonstrating that LILBID is ideally suited even to challenging samples.

While this shows that oligomeric states can be determined even for highly hydrophobic systems, this alone doesn't offer much structural information, which might be relevant for larger complexes.

**Figure 3.**
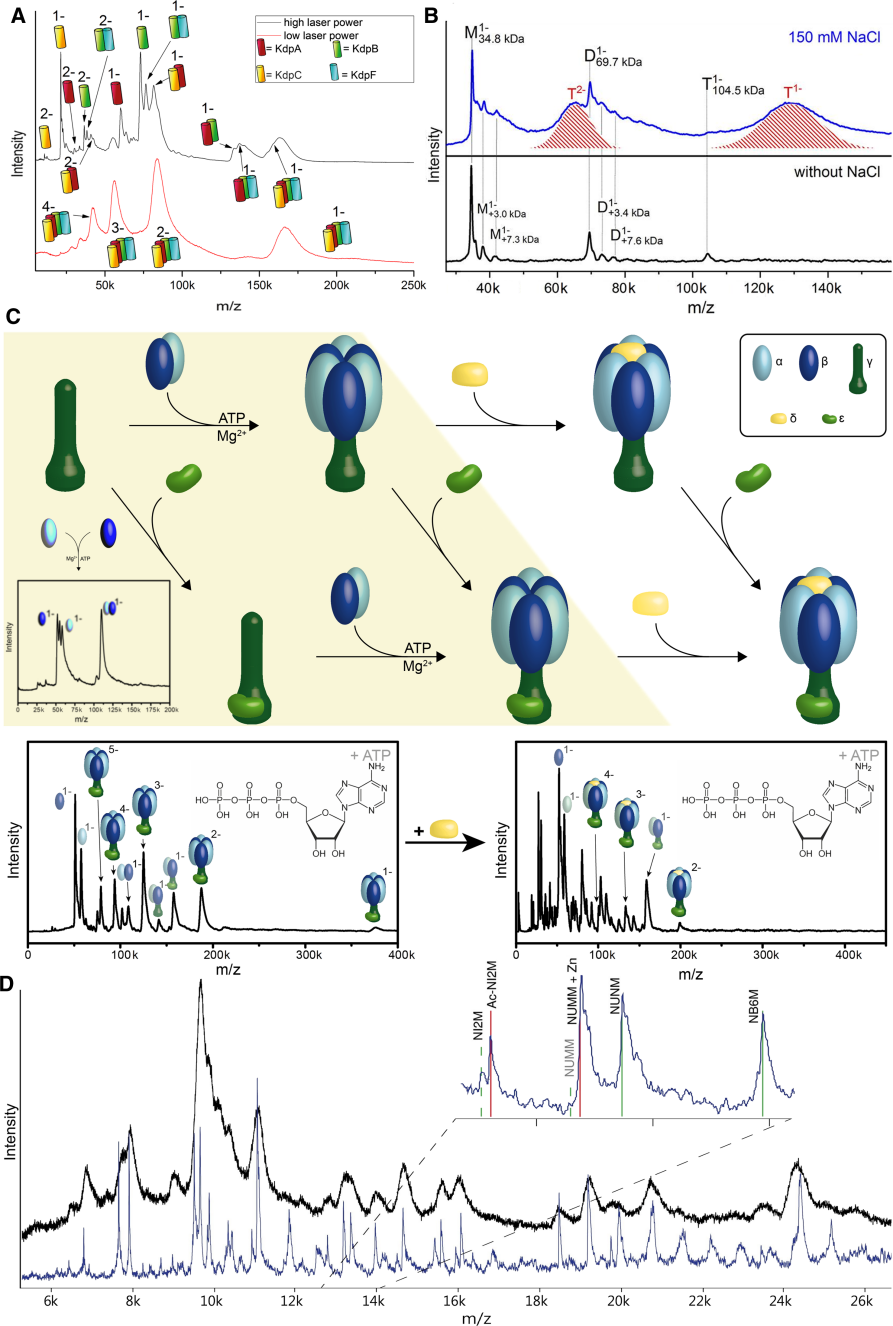
(**A**) LILBID spectra of KdpFABC at different laser power reveal the intact complex, as well as the subunits next neighbor relationships (unpublished). (**B**) LILBID spectra of purified AaHAS in the presence of 150 mM NaCl (top) and without NaCl (bottom). The red shadings in the upper spectrum represent the trimeric AaHAS with tightly bound lipids and salt. When this extra mass is missing the Trimer dissociates, showing their relevance for the complex stability [70]. (**C**) Formation pathway of the F_1_-ATPase investigated by LILBID. The yellow box indicates formation steps that are dependent on the presence of ATP and Mg cations. Left to right are subsequent steps while vertical formation steps are independent of previous complex formation [71]. (**D**) Subunit composition of Complex I from *Yarrowia lipolytica* recorded on LILBID first generation (black) [72] and second generation (blue). Increased mass resolving power and the use of a collision cell result in a much better resolution in the spectrum. The inset shows that the spectrum can reveal the mass shift of some subunits e.g. due to acylation or Zn binding (red vs. green lines) [72].

A useful feature of LILBID for such larger complexes is its potential to differentiate different binding interfaces. This can allow determination of structural aspects, which can be useful for homo as well as hetero oligomeric complexes. An example is the soluble Avidin homotetramer [17], for which the observed species were a dominant tetramer accompanied by dimeric and monomeric states in the complete absence of a trimer. These species give clear evidence that the tetramer is constructed as a dimer of a dimer. In contrast, this feature is not picked up in the nESI results, where the dominant species was the highly charged monomer created according to the chain-ejection model [35]. Tetramer and trimer are detected as very minor species, giving no evidence of the underlying structure of the complex. A more complex system is the K^+^ uptake system KdpFABC [67] shown in Figure 3A. LILBID spectra of this hetero-pentameric membrane complex allow at medium laser intensities some complex dissociation, while keeping the biologically relevant next-neighbor connections intact, as indicated by the pictograms in the spectrum. This reveals a set of subcomplexes, which give a clear idea of next neighbor relations and connectivities (Figure 3A). The same principle was utilized for the hetero-oligomeric GCD, a membrane protein complex consisting of four different proteins (GcdA, B, C and D) ([68, 69]). Using low laser power leads to the detection of the intact complex and large fragments of it, while high laser power shows mostly monomers and small complexes of subunits, revealing which are in contact to each other in the structure. Figure 3B shows the homo tetrameric membrane protein heme A synthase from *Aquifex aeolicus* (AaHAS) [70]. An interesting feature of the spectrum are the high mass shifts observed for the singly and doubly charged trimer (shaded in red), which are only observed in the presence of 150 mM NaCl. This mass shift includes lipids, which are lost, when the complex dissociates. Only a few lipids remain attached to the AaHAS proteins (grey lines), once the ring structure is opened, which suggests binding of lipids in the oligomerization interface. These findings allow the conclusion that lipids and salt may play an important role in the stabilization and assembly of the trimeric AaHAS complex.

In recent years interest regarding analysis of protein complexes generally moves beyond mere structural aspects and more emphasis is given towards understanding of the mechanisms, such as those controlling complexe formation. In a recent study the well-choreographed assembly process of an F-type ATPsynthase, a large multiprotein complex employed for ATP production, was investigated. LILBID was used to monitor the stepwise formation of the F_1_-complex and was the key tool to identify via which parallel pathways the individual subunits form essential building blocks and then the entire complex. The study as well identified which additives are needed in which complex formation step (shown in Figure 3C) and could show how the correct assembly correlates with the enzymes activity [71]. Similarly, LILBID could be employed to monitor the stepwise formation of an HSP90/kinase complex and to determine the previously unknown role on a PPIase in this process [73]. This approach is also suited to reveal changes in oligomeric states resulting from changes of the environment, as it was observed for the human TRPML2 ion channel at different pHs and PACE Transporter PA2880 from *P. aeruginosa* at different pHs [74, 75].

## Outlook

This review shows that alternative ionization mechanisms such as LILBID can be of high interest, as systems that are more complex are getting into the focus of structural biology. The realization that the understanding of the interplay in a cell requires more than just knowledge of the constituting parts, makes it increasingly clear, that future questions can only be understood with constantly improved methodology. This review gives an overview over requirements relevant for analysis of protein complexes and especially membrane protein complexes with their special demands, which can be met by LILBID-MS applications. Additionally applications of LILBID analysis are shown, which go beyond mere determination of mass, such as understanding of a complex assembly pathway. Improving the possible resolution of the mass spectra recorded using LILBID should further increase the applicability of LILBID as it allows e.g. for the determination of post-translational modifications (PTMs) top-down out of a complex. An example for this is shown in Figure 3D, which shows one of the first spectra gained with a LILBID ion source in combination with a commercially available instrument (Micromass QTof). Coupling a LILBID ion source with commercially available instruments will in the future allow for better resolved mass spectra as well as the combined use of LILBID with modern orthogonal analytic methods e.g. ion mobility spectrometry.

## Perspectives

- Alternative ionization mechanisms for native mass spectrometry are of high interest, as questions to be answered in structural biology are increasing in complexity. Orthogonal features of such analytical methods can open the road to the investigation of more complex systems.
- Native MS has established itself as a routine tool to investigate a variety of biological samples and has gone beyond just being a method that determines the weight of a molecule. LILBID-MS has shown to be especially useful for certain substance classes, such as membrane complexes, where it can be difficult to analyze them with the commercially available methods, especially when novel microbiological methods are used.
- Aspects which will be further developed in the near future for LILBID are determination of binding affinities of protein complexes and their subunits and continuing down the road of improved membrane-like environments with the goal of analyzing the membrane protein complex directly out of the cell membrane.

## Abbreviations

AaHAS: heme A synthase from *Aquifex aeolicus*
LILBID: laser induced liquid bead ion desorption
MS: mass spectrometry
MSP: membrane scaffold protein
PTMs: post-translational modifications
SID: surface induced dissociation
SMA: styrene-maleic acid
SMALPs: SMA lipid particles

## References

[1] Bender, J. and Schmidt, C. (2019) Mass spectrometry of membrane protein complexes. Biol. Chem. 400, 813–829 10.1515/hsz-2018-0443 30956223

[2] Frick, M. and Schmidt, C. (2019) Mass spectrometry-A versatile tool for characterising the lipid environment of membrane protein assemblies. Chem. Phys. Lipids 221, 145–157 10.1016/j.chemphyslip.2019.04.001 30953608

[3] Sahin, C., Reid, D.J., Marty, M.T. and Landreh, M. (2020) Scratching the surface: native mass spectrometry of peripheral membrane protein complexes. Biochem. Soc. Trans. 48, 547–558 10.1042/BST20190787 32129823 PMC7192793

[4] Agasid, M.T. and Robinson, C.V. (2021) Probing membrane protein-lipid interactions. Curr. Opin. Struct. Biol. 69, 78–85 10.1016/j.sbi.2021.03.010 33930613

[5] Allison, T.M. and Bechara, C. (2019) Structural mass spectrometry comes of age: new insight into protein structure, function and interactions. Biochem. Soc. Trans. 47, 317–327 10.1042/BST20180356 30647140

[6] Bennett, J.L., Nguyen, G.T.H. and Donald, W.A. (2022) Protein-small molecule interactions in native mass spectrometry. Chem. Rev. 122, 7327–7385 10.1021/acs.chemrev.1c00293 34449207

[7] Bolla, J.R., Fiorentino, F. and Robinson, C.V. (2021) Mass spectrometry informs the structure and dynamics of membrane proteins involved in lipid and drug transport. Curr. Opin. Struct. Biol. 70, 53–60 10.1016/j.sbi.2021.03.014 33964676

[8] Karch, K.R., Snyder, D.T., Harvey, S.R. and Wysocki, V.H. (2022) Native mass spectrometry: recent progress and remaining challenges. Annu. Rev. Biophys. 51, 157–179 10.1146/annurev-biophys-092721-085421 34982572 PMC10700022

[9] Tamara, S., den Boer, M.A. and Heck, A.J.R. (2022) High-Resolution native mass spectrometry. Chem. Rev. 122, 7269–7326 10.1021/acs.chemrev.1c00212 34415162 PMC9052423

[10] van Schaick, G., Haselberg, R., Somsen, G.W., Wuhrer, M. and Domínguez-Vega, E. (2022) Studying protein structure and function by native separation–mass spectrometry. Nat. Rev. Chem. 6, 215–231 10.1038/s41570-021-00353-7 37117432

[11] Webb, I.K. (2022) Recent technological developments for native mass spectrometry. Biochim. Biophys. Acta 1870, 140732 10.1016/j.bbapap.2021.140732 PMC9219587 34653668

[12] Zhou, M., Lantz, C., Brown, K.A., Ge, Y., Paša-Tolić, L., Loo, J.A. et al. (2020) Higher-order structural characterisation of native proteins and complexes by top-down mass spectrometry. Chem. Sci. 11, 12918–12936 10.1039/D0SC04392C 34094482 PMC8163214

[13] Keener, J.E., Zhang, G. and Marty, M.T. (2021) Native mass spectrometry of membrane proteins. Anal. Chem. 93, 583–597 10.1021/acs.analchem.0c04342 33115234 PMC7855921

[14] Rolland, A.D. and Prell, J.S. (2022) Approaches to heterogeneity in native mass spectrometry. Chem. Rev 122, 7909–7951 10.1021/acs.chemrev.1c00696 34470219 PMC8885964

[15] Morgner, N., Barth, H.-D. and Brutschy, B. (2006) A new way to detect noncovalently bonded complexes of biomolecules from liquid micro-droplets by laser mass spectrometry. Aust. J. Chem. 59, 109 10.1071/CH05285

[16] Morgner, N., Kleinschroth, T., Barth, H.-D., Ludwig, B. and Brutschy, B. (2007) A novel approach to analyze membrane proteins by laser mass spectrometry: from protein subunits to the integral complex. J. Am. Soc. Mass Spectrom. 18, 1429–1438 10.1016/j.jasms.2007.04.013 17544294

[17] Peetz, O., Hellwig, N., Henrich, E., Mezhyrova, J., Dötsch, V., Bernhard, F. et al. (2019) LILBID and nESI: different native mass spectrometry techniques as tools in structural biology. J. Am. Soc. Mass Spectrom. 30, 181–191 10.1007/s13361-018-2061-4 30225732 PMC6318263

[18] Brandt, K., Müller, D.B., Hoffmann, J., Langer, J.D., Brutschy, B., Morgner, N. et al. (2016) Stoichiometry and deletion analyses of subunits in the heterotrimeric F-ATP synthase c ring from the acetogenic bacterium *Acetobacterium woodii*. FEBS J. 283, 510–520 10.1111/febs.13606 26613566

[19] Young, P., Hense, G., Immer, C., Wöhnert, J. and Morgner, N. (2020) LILBID laser dissociation curves: a mass spectrometry-based method for the quantitative assessment of dsDNA binding affinities. Sci. Rep. 10, 20398 10.1038/s41598-020-76867-9 33230224 PMC7683618

[20] Warschat, C., Stindt, A., Panne, U. and Riedel, J. (2015) Mass spectrometry of levitated droplets by thermally unconfined infrared-laser desorption. Anal. Chem. 87, 8323–8327 10.1021/acs.analchem.5b01495 26165504

[21] Asami, H., Kawabata, R., Kawauchi, N. and Kohno, J. (2019) Photodissociation spectroscopy of hydrated myoglobin ions isolated by IR-laser ablation of a droplet beam: recovery from pH-denatured structure by gas-phase isolation. Chem. Lett. 48, 140–143 10.1246/cl.180884

[22] Kohno, J., Nabeta, K. and Sasaki, N. (2013) Charge state of lysozyme molecules in the gas phase produced by IR-laser ablation of droplet beam. J. Phys. Chem. A 117, 9–14 10.1021/jp3096506 23234475

[23] Michalik-Onichimowska, A., Beitz, T., Panne, U., Löhmannsröben, H.-G. and Riedel, J. (2017) Microsecond mid-infrared laser pulses for atmospheric pressure laser ablation/ionization of liquid samples. Sens. Actuators B Chem. 238, 298–305 10.1016/j.snb.2016.06.155

[24] Pirkl, A., Soltwisch, J., Draude, F. and Dreisewerd, K. (2012) Infrared matrix-assisted laser desorption/ionization orthogonal-time-of-flight mass spectrometry employing a cooling stage and water ice as a matrix. Anal. Chem. 84, 5669–5676 10.1021/ac300840b 22670870

[25] Schulze, S., Pahl, M., Stolz, F., Appun, J., Abel, B., Schneider, C. et al. (2017) Liquid beam desorption mass spectrometry for the investigation of continuous flow reactions in microfluidic chips. Anal. Chem. 89, 6175–6181 10.1021/acs.analchem.7b01026 28489359

[26] Susa, A.C., Xia, Z. and Williams, E.R. (2017) Native mass spectrometry from common buffers with salts that mimic the extracellular environment. Angew. Chem. 129, 8020–8023 10.1002/ange.201702330 PMC12711215 28510995

[27] Hernández, H. and Robinson, C.V. (2007) Determining the stoichiometry and interactions of macromolecular assemblies from mass spectrometry. Nat. Protoc. 2, 715–726 10.1038/nprot.2007.73 17406634

[28] Wang, G. and Cole, R.B. (1994) Effect of solution ionic strength on analyte charge state distributions in positive and negative Ion electrospray mass spectrometry. Anal. Chem. 66, 3702–3708 10.1021/ac00093a026

[29] Toyoshima, C. and Nomura, H. (2002) Structural changes in the calcium pump accompanying the dissociation of calcium. Nature 418, 605–611 10.1038/nature00944 12167852

[30] Konermann, L. (2017) Addressing a common misconception: ammonium acetate as neutral pH “Buffer” for native electrospray mass spectrometry. J. Am. Soc. Mass Spectrom. 28, 1827–1835 10.1007/s13361-017-1739-3 28710594

[31] Loo, R.R., Dales, N. and Andrews, P.C. (1996) The effect of detergents on proteins analyzed by electrospray ionization. Methods Mol. Biol. 61, 141–160 10.1385/0-89603-345-7:141 8930871

[32] Zhou, M. and Wysocki, V.H. (2014) Surface induced dissociation: dissecting noncovalent protein complexes in the gas phase. Acc. Chem. Res. 47, 1010–1018 10.1021/ar400223t 24524650

[33] Stiving, A.Q., VanAernum, Z.L., Busch, F., Harvey, S.R., Sarni, S.H. and Wysocki, V.H. (2019) Surface-induced dissociation: an effective method for characterization of protein quaternary structure. Anal. Chem. 91, 190–209 10.1021/acs.analchem.8b05071 30412666 PMC6571034

[34] Snyder, D.T., Harvey, S.R. and Wysocki, V.H. (2022) Surface-induced dissociation mass spectrometry as a structural biology tool. Chem. Rev. 122, 7442–7487 10.1021/acs.chemrev.1c00309 34726898 PMC9282826

[35] Konermann, L., Ahadi, E., Rodriguez, A.D. and Vahidi, S. (2013) Unraveling the mechanism of electrospray ionization. Anal. Chem. 85, 2–9 10.1021/ac302789c 23134552

[36] Saikusa, K., Kato, D., Nagadoi, A., Kurumizaka, H. and Akashi, S. (2020) Native mass spectrometry of protein and DNA complexes prepared in nonvolatile buffers. J. Am. Soc. Mass Spectrom. 31, 711–718 10.1021/jasms.9b00145 31999114

[37] Flick, T.G., Cassou, C.A., Chang, T.M. and Williams, E.R. (2012) Solution additives that desalt protein ions in native mass spectrometry. Anal. Chem. 84, 7511–7517 10.1021/ac301629s 22881839 PMC3433631

[38] Susa, A.C., Lippens, J.L., Xia, Z., Loo, J.A., Campuzano, I.D.G. and Williams, E.R. (2018) Submicrometer emitter ESI tips for native mass spectrometry of membrane proteins in ionic and nonionic detergents. J. Am. Soc. Mass Spectrom. 29, 203–206 10.1007/s13361-017-1793-x 29027132 PMC5786471

[39] Susa, A.C., Xia, Z. and Williams, E.R. (2017) Native mass spectrometry from common buffers with salts that mimic the extracellular environment. Angew. Chem. Int. Ed. Engl. 56, 7912–7915 10.1002/anie.201702330 28510995 PMC12711215

[40] Susa, A.C., Xia, Z. and Williams, E.R. (2017) Small emitter tips for native mass spectrometry of proteins and protein complexes from nonvolatile buffers that mimic the intracellular environment. Anal. Chem. 89, 3116–3122 10.1021/acs.analchem.6b04897 28192954

[41] Xia, Z., DeGrandchamp, J.B. and Williams, E.R. (2019) Native mass spectrometry beyond ammonium acetate: effects of nonvolatile salts on protein stability and structure. Analyst 144, 2565–2573 10.1039/C9AN00266A 30882808

[42] Diskowski, M., Mehdipour, A.R., Wunnicke, D., Mills, D.J., Mikusevic, V., Bärland, N. et al. (2017) Helical jackknives control the gates of the double-pore K+ uptake system KtrAB. eLife 6, e24303 10.7554/eLife.24303 28504641 PMC5449183

[43] Bayburt, T.H., Grinkova, Y.V. and Sligar, S.G. (2002) Self-Assembly of discoidal phospholipid bilayer nanoparticles with membrane scaffold proteins. Nano Lett. 2, 853–856 10.1021/nl025623k

[44] Denisov, I.G. and Sligar, S.G. (2016) Nanodiscs for structural and functional studies of membrane proteins. Nat. Struct. Mol. Biol. 23, 481–486 10.1038/nsmb.3195 27273631 PMC8934039

[45] Denisov, I.G. and Sligar, S.G. (2017) Nanodiscs in membrane biochemistry and biophysics. Chem. Rev. 117, 4669–4713 10.1021/acs.chemrev.6b00690 28177242 PMC5805400

[46] Li, J., Richards, M.R., Kitova, E.N. and Klassen, J.S. (2017) Delivering transmembrane peptide complexes to the gas phase using nanodiscs and electrospray ionization. J. Am. Soc. Mass Spectrom. 28, 2054–2065 10.1007/s13361-017-1735-7 28681358

[47] Marty, M.T., Baldwin, A.J., Marklund, E.G. Hochberg, G.K.A., Benesch, J.L.P. and Robinson, C.V. (2015) Bayesian deconvolution of mass and ion mobility spectra: from binary interactions to polydisperse ensembles. Anal. Chem. 87, 4370–4376 10.1021/acs.analchem.5b00140 25799115 PMC4594776

[48] Marty, M.T., Zhang, H., Cui, W., Gross, M.L. and Sligar, S.G. (2014) Interpretation and deconvolution of nanodisc native mass spectra. J. Am. Soc. Mass Spectrom. 25, 269–277 10.1007/s13361-013-0782-y 24353133 PMC3918181

[49] Kostelic, M.M., Zak, C.K., Jayasekera, H.S. and Marty, M.T. (2021) Assembly of model membrane nanodiscs for native mass spectrometry. Anal. Chem. 93, 5972–5979 10.1021/acs.analchem.1c00735 33797873

[50] Zhang, G., Keener, J.E. and Marty, M.T. (2020) Measuring remodeling of the lipid environment surrounding membrane proteins with lipid exchange and native mass spectrometry. Anal. Chem. 92, 5666–5669 10.1021/acs.analchem.0c00786 32250609 PMC7204402

[51] Marty, M.T., Hoi, K.K., Gault, J. and Robinson, C.V. (2016) Probing the lipid annular belt by gas-phase dissociation of membrane proteins in nanodiscs. Angew. Chem. Int. Ed. Engl. 55, 550–554 10.1002/anie.201508289 26594028 PMC4736441

[52] Henrich, E., Sörmann, J., Eberhardt, P., Peetz, O., Mezhyrova, J., Morgner, N. et al. (2017) From gene to function: cell-free electrophysiological and optical analysis of ion pumps in nanodiscs. Biophys. J. 113, 1331–1341 10.1016/j.bpj.2017.03.026 28450130 PMC5607034

[53] Waberer, L., Henrich, E., Peetz, O., Morgner, N., Dötsch, V., Bernhard, F. et al. (2017) The synaptic vesicle protein SV31 assembles into a dimer and transports Zn2. J. Neurochem. 140, 280–293 10.1111/jnc.13886 27917477

[54] Henrich, E., Löhr, F., Pawlik, G., Peetz, O., Dötsch, V., Morgner, N. et al. (2018) Lipid conversion by cell-free synthesized phospholipid methyltransferase Opi3 in defined nanodisc membranes supports an in trans mechanism. Biochemistry 57, 5780–5784 10.1021/acs.biochem.8b00807 30226041

[55] Henrich, E., Peetz, O., Hein, C., Laguerre, A., Hoffmann, B., Hoffmann, J. et al. (2017) Analyzing native membrane protein assembly in nanodiscs by combined non-covalent mass spectrometry and synthetic biology. eLife 6, e20954 10.7554/eLife.20954 28067619 PMC5291076

[56] Peetz, O., Henrich, E., Laguerre, A., Löhr, F., Hein, C., Dötsch, V. et al. (2017) Insights into cotranslational membrane protein insertion by combined LILBID-mass spectrometry and NMR spectroscopy. Anal. Chem. 89, 12314–8 10.1021/acs.analchem.7b03309 29039652

[57] Klyszejko, A.L., Shastri, S., Mari, S.A., Grubmüller, H., Muller, D.J. and Glaubitz, C. (2008) Folding and assembly of proteorhodopsin. J. Mol. Biol. 376, 35–41 10.1016/j.jmb.2007.11.030 18155728

[58] Hellwig, N., Peetz, O., Ahdash, Z., Tascón, I., Booth, P.J., Mikusevic, V. et al. (2018) Native mass spectrometry goes more native: investigation of membrane protein complexes directly from SMALPs. Chem. Commun. (Camb) 54, 13702–5 10.1039/C8CC06284F 30452022 PMC6289172

[59] Jamshad, M., Lin, Y.-P., Knowles, T.J., Parslow, R.A., Harris, C., Wheatley, M. et al. (2011) Surfactant-free purification of membrane proteins with intact native membrane environment. Biochem. Soc. Trans. 39, 813–818 10.1042/BST0390813 21599653

[60] Knowles, T.J., Finka, R., Smith, C., Lin, Y.-P., Dafforn, T. and Overduin, M. (2009) Membrane proteins solubilized intact in lipid containing nanoparticles bounded by styrene maleic acid copolymer. J. Am. Chem. Soc. 131, 7484–7485 10.1021/ja810046q 19449872

[61] Simon, K.S., Pollock, N.L. and Lee, S.C. (2018) Membrane protein nanoparticles: the shape of things to come. Biochem. Soc. Trans. 46, 1495–1504 10.1042/BST20180139 30464048 PMC6299238

[62] Hoi, K.K., Bada Juarez, J.F., Judge, P.J., Yen, H.-Y., Wu, D., Vinals, J. et al. (2021) Detergent-free lipodisq nanoparticles facilitate high-resolution mass spectrometry of folded integral membrane proteins. Nano Lett. 21, 2824–2831 10.1021/acs.nanolett.0c04911 33787280 PMC8050825

[63] Frauenfeld, J., Löving, R., Armache, J.-P., Sonnen, A.F.-P., Guettou, F., Moberg, P. et al. (2016) A saposin-lipoprotein nanoparticle system for membrane proteins. Nat. Methods 13, 345–351 10.1038/nmeth.3801 26950744 PMC4894539

[64] Flayhan, A., Mertens, H.D., Ural-Blimke, Y., Martinez Molledo, M., Svergun, D.I. and Löw, C. (2018) Saposin lipid nanoparticles: a highly versatile and modular tool for membrane protein research. Structure 26, 345–355.e5 10.1016/j.str.2018.01.007 29413323 PMC5807053

[65] Benesch, J.L.P., Sobott, F. and Robinson, C.V. (2003) Thermal dissociation of multimeric protein complexes by using nanoelectrospray mass spectrometry. Anal. Chem. 75, 2208–2214 10.1021/ac034132x 12918957

[66] Jurchen, J.C. and Williams, E.R. (2003) Origin of asymmetric charge partitioning in the dissociation of gas-phase protein homodimers. J. Am. Chem. Soc. 125, 2817–2826 10.1021/ja0211508 12603172 PMC1325210

[67] Stock, C., Hielkema, L., Tascón, I., Wunnicke, D., Oostergetel, G.T., Azkargorta, M. et al. (2018) Cryo-EM structures of KdpFABC suggest a K^+^ transport mechanism via two inter-subunit half-channels. Nat. Commun. 9, 4971 10.1038/s41467-018-07319-2 30478378 PMC6255902

[68] Braune, A., Bendrat, K., Rospert, S. and Buckel, W. (1999) The sodium ion translocating glutaconyl-CoA decarboxylase from *Acidaminococcus fermentans*: cloning and function of the genes forming a second operon. Mol. Microbiol. 31, 473–487 10.1046/j.1365-2958.1999.01189.x 10027965

[69] Vitt, S., Prinz, S., Hellwig, N., Morgner, N., Ermler, U. and Buckel, W. (2020) Molecular and low-resolution structural characterization of the Na^+^-translocating glutaconyl-CoA decarboxylase from clostridium symbiosum. Front. Microbiol. 11, 480 10.3389/fmicb.2020.00480 32300335 PMC7145394

[70] Zeng, H., Zhu, G., Zhang, S., Li, X., Martin, J., Morgner, N. et al. (2020) Isolated heme A synthase from aquifex aeolicus is a trimer. mBio 11, e02615-19 10.1128/mBio.02615-19 32605991 PMC7327177

[71] Huu K, V., Zangl, R., Hoffmann, J., Just, A. and Morgner, N. (2022) Bacterial F-type ATP synthases follow a well-choreographed assembly pathway. Nat. Commun. 13, 1218 10.1038/s41467-022-28828-1 35260553 PMC8904574

[72] Morgner, N., Zickermann, V., Kerscher, S., Wittig, I., Abdrakhmanova, A., Barth, H.-D. et al. (2008) Subunit mass fingerprinting of mitochondrial complex I. Biochim. Biophys. Acta 1777, 1384–1391 10.1016/j.bbabio.2008.08.001 18762163

[73] Sima, S., Barkovits, K., Marcus, K., Schmauder, L., Hacker, S.M., Hellwig, N. et al. (2021) HSP-90/kinase complexes are stabilized by the large PPIase FKB-6. Sci. Rep. 11, 12347 10.1038/s41598-021-91667-5 34117308 PMC8196007

[74] Viet, K.K., Wagner, A., Schwickert, K., Hellwig, N., Brennich, M., Bader, N. et al. (2019) Structure of the human TRPML2 ion channel extracytosolic/lumenal domain. Structure 27, 1246–1257.e5 10.1016/j.str.2019.04.016 31178222

[75] Zhao, J., Hellwig, N., Djahanschiri, B., Khera, R., Morgner, N., Ebersberger, I. et al. (2022) Assembly and functional role of PACE transporter PA2880 from *Pseudomonas aeruginosa*. Microbiol. Spectr. 10, e0145321 10.1128/spectrum.01453-21 35377188 PMC9045395

